# A Developmental Perspective on Paragangliar Tumorigenesis

**DOI:** 10.3390/cancers11030273

**Published:** 2019-02-26

**Authors:** Lavinia Vittoria Lotti, Simone Vespa, Mattia Russel Pantalone, Silvia Perconti, Diana Liberata Esposito, Rosa Visone, Angelo Veronese, Carlo Terenzio Paties, Mario Sanna, Fabio Verginelli, Cecilia Soderberg Nauclér, Renato Mariani-Costantini

**Affiliations:** 1 Department of Experimental Medicine, “La Sapienza” University, Viale Regina Elena 324, 00161 Rome, Italy; laviniavittoria.lotti@uniroma1.it; 2 Center of Sciences on Aging and Translational Medicine (CeSI-MeT), “G. d’Annunzio” University, Via Luigi Polacchi 11, 66100 Chieti, Italy; sv85@libero.it (S.V.); percontisilvia@gmail.com (S.P.); d.esposito@unich.it (D.L.E); r.visone@unich.it (R.V.); 3 Department of Medical, Oral and Biotechnological Sciences, “G. d’Annunzio” University, Via dei Vestini 31, 66100 Chieti, Italy; 4 Department of Medicine (Solna), Division of Microbial Pathogenesis, BioClinicum, Karolinska Institutet, 17164 Stockholm, Sweden; mattia.pantalone@ki.se (M.R.P.); cecilia.naucler@ki.se (C.S.N.); 5 Department of Medicine and Aging Sciences, “G. d’Annunzio” University, Via Luigi Polacchi 11, 66100 Chieti, Italy; a.veronese@unich.it; 6 Department of Oncology-Hematology, Service of Anatomic Pathology, “Guglielmo da Saliceto” Hospital, Via Taverna 49, 29100 Piacenza, Italy; carlopaties@yahoo.it; 7 Skull Base Unit, “Gruppo Otologico” Piacenza-Roma, Via Antonio Emmanueli, 42, 29121 Piacenza, Italy; mario.sanna@gruppootologico.it; 8 Department of Pharmacy, “G. d’Annunzio” University, Via dei Vestini 31, 66100 Chieti, Italy; verginelli@unich.it

**Keywords:** carotid body, angiogenesis, mitochondria, neural crest, neurogenesis, paraganglioma, stem-like tumor cells, vasculogenesis, xenograft

## Abstract

In this review, we propose that paraganglioma is a fundamentally organized, albeit aberrant, tissue composed of neoplastic vascular and neural cell types that share a common origin from a multipotent mesenchymal-like stem/progenitor cell. This view is consistent with the pseudohypoxic footprint implicated in the molecular pathogenesis of the disease, is in harmony with the neural crest origin of the paraganglia, and is strongly supported by the physiological model of carotid body hyperplasia. Our immunomorphological and molecular studies of head and neck paragangliomas demonstrate in all cases relationships between the vascular and the neural tumor compartments, that share mesenchymal and immature vasculo-neural markers, conserved in derived cell cultures. This immature, multipotent phenotype is supported by constitutive amplification of NOTCH signaling genes and by loss of the microRNA-200s and -34s, which control *NOTCH1*, *ZEB1*, and *PDGFRA* in head and neck paraganglioma cells. Importantly, the neuroepithelial component is distinguished by extreme mitochondrial alterations, associated with collapse of the ΔΨm. Finally, our xenograft models of head and neck paraganglioma demonstrate that mesenchymal-like cells first give rise to a vasculo-angiogenic network, and then self-organize into neuroepithelial-like clusters, a process inhibited by treatment with imatinib.

## 1. Introduction

### 1.1. Intersections between Tumorigenesis, Histogenesis, and Tissue Regeneration

Tumors are capable of autonomous and aberrant growth, but, as normal tissues, can grow only after achieving a structural organization, which requires the coordinated contribution of different cell types, the establishment of appropriate cell–cell and cell–matrix interactions and the development of specific scaffolds and vascular networks [1]. However, much of the basic information about the structural and functional organization of neoplastic tissues is still lacking. For instance, the key question of whether tumors contain cells able to transdifferentiate into both vascular and parenchymal cell types is still debated [2]. We do not know to what extent tumors follow the histogenetic blueprint of their normal tissue counterparts, and we do not fully understand which of the several tumor-resident cell types can regenerate neoplastic tissue after damage inflicted by therapy [3,4,5,6]. Nonetheless, it is clear that evolutionarily conserved developmental programs and signaling pathways intersect tumorigenesis, histo/organogenesis, and tissue repair/regeneration [1,6]. In particular, invasive and/or metastatic tumors essentially imitate the organogenetic program of the neural crest, a transient embryonic structure that characterizes the evolution of procraniates and craniates (*Cristozoa*) [7]. The temporary neural crest milieu defines a highly plastic population of migratory and multipotent cells that, in response to complex signals—including morphogen gradients, cell–cell interactions, availability of oxygen and nutrients, and topography—dedifferentiate via the epithelial–mesenchymal transition (EMT) program, migrate, proliferate, and again re-differentiate via the reverse mesenchymal–epithelial transition (MET) program, giving rise to an amazing variety of cell types and tissues throughout the axial body region [4,8]. While the embryonic population of neural crest cells is ephemeral, it appears that in postnatal tissues and organs the perivascular niche preserves multipotent stem/progenitor-like cells that retain tissue-specific histogenetic instructions that are reactivated during regeneration and repair [4,9,10,11,12]. Such cells might link development, tissue regeneration, and neoplasia.

### 1.2. Paragangliomas and Pheochromocytomas

Paragangliomas (PGLs) are rare, generally sluggish but invasive and potentially lethal tumors arising from the neural crest-derived paraxial autonomic ganglia (paraganglia) of parasympathetic (mainly head and neck) or sympathoadrenal (mainly truncal) lineage [13]. Pheochromocytomas are in essence catecholamine-producing tumors that arise mainly from the chromaffin cells of the adrenal medulla, also of neural crest origin, and present with a constellation of symptoms secondary to catecholamine overload, eventually leading to severe cardiovascular disorders and death [14]. It is estimated that 10–20% of all pheochromocytomas and PGLs (collectively termed PPGLs) manifest a malignant behavior, in terms of synchronous or metachronous metastatic spread, generally associated with poor prognosis [15]. Metastatic progression seems less common in head and neck PGLs (HNPGL, ≈5%) and pheochromocytomas (≈10%) than in thoraco-abdominal PGLs (15% to 35%) [15,16,17]. However, despite intensive research, no clinicopathological, molecular, or genetic criteria that unequivocally distinguish PPGLs with metastatic potential have been identified [15,16,17,18]. Therefore, to overcome diagnostic problems, the WHO Endocrine Tumor Classification recently acknowledged metastatic potential to all PPGLs [19,20]. This implies life-long follow-up after surgery for all cases and additional risk stratification according to pathological, clinical, biochemical, and genetic evidence [17,21].

**Figure 1:**
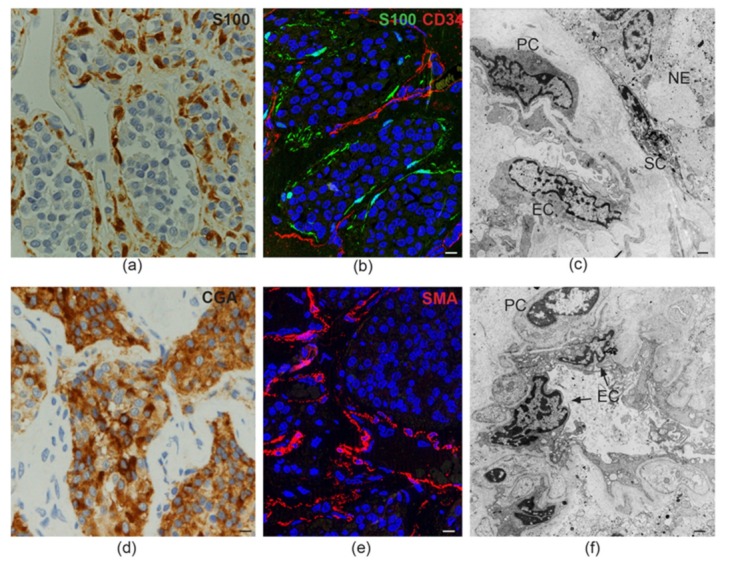
Tissue-like organization in paraganglioma. (**a**) Dark brown immunohistochemical staining for S100, a glial marker, highlights the sustentacular cell component surrounding the alveolar nests (“zellballen”) of neuroepithelial (“chief”) cells (avidin-biotin immunoperoxidase counterstained with hematoxylin and eosin, bar = 10 µm). (**b**) Immunofluorescence highlights thin sustentacular cells at the edges of neuroepithelial “zellballen,” identified by labeling with antibody to S100 (green). The endothelial lining of the capillaries in the surrounding stroma is labeled in red with CD34 (double immunofluorescence on semithin frozen section, bar = 10 µm). (**c**) Transmission electron micrograph showing the cytological features and topological relationships of the four main cell types that organize the paraganglioma microenvironment (endothelial cells, EC; pericytes, PC; sustentacular cells, SC; neuroepithelial cells, NE). Note the nuclear pleomorphism and similarities between chromatin patterns (bar = 2 µm). (**d**) Dark brown cytoplasmic staining for chromogranin A (CGA), a marker for neuroendocrine neoplasia, highlights neuroepithelial cell nests (“zellballen”) (avidin-biotin immunoperoxidase counterstained with hematoxylin and eosin, bar = 10 µm). (**e**) Immunofluorescence highlights the pericytic/mural cell component of the paraganglioma vasculature, identified by red labeling with antibody to smooth muscle actin (SMA, immunofluorescence on semithin frozen section, bar = 10 µm). (**f**) Ultrastructural cross section of a paraganglioma capillary showing the atypical cytological features of endothelial cells (EC) and pericytes (PC), two cell types whose roles in paragangliar tumorigenesis have been thus far scarcely considered (bar = 2 µm).

Collectively, PPGLs may provide important insights into the intersection(s) between organogenesis and tumorigenesis, as it is plainly evident that their basically conserved histostructure mimics that shared by their normal tissue counterparts, the extramedullary paraganglia and the adrenal medulla. In fact, as exemplified in Figure 1, PPGL tissue quite invariably consists in nests or ribbons of more or less dysplastic neurosecretory cells, fairly circumscribed and “nursed” by glial cells, with the whole resting on a highly vascular framework composed of dysplastic endothelia and pericytes that may assume frankly angiomatous features [22]. Thus, PPGLs provide a model for “organoid” tumors, i.e., tumors consisting of a tridimensional assemblage of cells of more than one type, arranged to form predictable tissue-like structures mimicking those of the organ of origin.

Intriguingly, PPGLs are among the tumors most frequently associated with autosomal dominant genetic predisposition, found in up to ≈40% of the cases [23,24,25]. The genes most commonly involved are those encoding the four subunits of the succinate dehydrogenase (SDH) enzyme, namely *SDHA*, *SDHB*, *SDHC*, and *SDHD*, and the SDH assembly co-factor, i.e., *SDHAF2*. Furthermore, PPGLs have been associated with germline mutations in other genes, including *RET*, *NF1*, *VHL*, *EPAS1*, *FH*, *MDH2*, *EGLN1/2*, *TMEM127*, and *MAX*, some of which are linked to hereditary neoplastic syndromes including other neural crest tumors, such as multiple endocrine neoplasia (*RET*), von-Hippel-Lindau syndrome (*VHL*), neurofibromatosis type 1 (*NF1*), and Carney–Stratakis syndrome (*SDH* genes) [23,24,25]. Notably, a maternal parent-of-origin effect, interpreted as evidence for “imprinting,” is implicated in the transmission of *SDHD*, *SDHAF2*, and *MAX* mutations [26]. Regardless of this effect, which may result in generation skipping, the penetrance of the mutations in the *SDH* genes that are most commonly associated with PPGL is surprisingly low; in fact, it has been reliably estimated at only 1.7% for *SDHA*, 22.0% for *SDHB*, and 8.3% for *SDHC* [27]. Furthermore, mice mutated in *sdhb*, the human *SDHB* homolog, do not develop any type of cancer [28]. All this suggests that germline *SDH* mutations predispose to PPGL, but are not sufficient for tumorigenesis. The environmental and/or constitutional factors that might modulate hereditary PPGL risk and contribute to PPGL, even in the absence of genetic predisposition, are currently unknown, with the exception, for carotid body PGL, of exposure to chronic hypoxia, such as in people living at high altitudes or in patients affected with chronic obstructive pulmonary disease or cyanotic heart defects [29,30,31,32].

Importantly, the most relevant genes implicated in PPGL predisposition, namely the *SDH* genes and *VHL*, as well as *EPAS1*, *FH*, *MDH2*, and *EGLN1/2*, link PPGL tumorigenesis to pseudohypoxia, a cellular phenotype characterized by the constitutive expression of proteins involved in the adaptive responses to low partial pressures of oxygen [23,24,25]. Among other pleiotropic effects on metabolism, the EMT, vasculoangiogenesis, etc., pseudohypoxia deregulates growth factor signaling and attenuates cell death, promoting the expansion of immature cell populations [33]. The same processes, induced to various extents by chronic environmental hypoxia, are implicated in the adaptive growth of the carotid body, the paraganglion at the basis of the homeostatic oxygen-sensing system. Notably, the carotid body is the most frequent site of origin of head and neck PGL (HNPGL) [34].

## 2. The Physiological Model of Carotid Body Hyperplasia Under Chronic Hypoxia May Illuminate Paraganglioma Development

Carotid body development has been recently delineated in a notable series of elegant studies from Ricardo Pardal’s group [35,36,37]. The carotid body is implicated in the organismal adaptation to chronic hypoxia, as in people living at high altitudes or in patients with cardiorespiratory diseases, in which cases, this organelle sustains marked hyperplasia and hypertrophy, reflecting the combined expansion of the neural and vascular tissue components, as in PGL. Pardal’s lab has clearly shown that this adaptive process is made possible via hypoxia inducible factor (HIF)-dependent reactivation of neural crest-derived resident stem-like cells retaining mesectodermal differentiation potential [36,37]. Such cells, overlooked because of lack of distinctive markers, remain quiescent under normoxia, but, under low partial pressure of oxygen, acquire a nestin +/GFAP- stem/progenitor cell phenotype and convert not only into new sustentacular and neuroepithelial cells, but also into endothelial and pericytic/mural cells, thus contributing to the impressive vasculoangiogenesis that sustains the hyperplastic carotid body. This capability of vasculo/neural transdifferentiation is consistent with the fact that both the neural ganglia of the autonomic nervous system and the cardiovascular structures of the upper trunk originate from the cephalic neural crest during embryogenesis [36]. Furthermore, it has been shown that stem-like neural cells can convert into vascular cells in vitro, and that neoplastic stem-like cells from neural tumors, such as glioblastoma, can give rise to tumor-derived endothelia in immunodeficient mice. This process, defined as vasculogenic mimicry, rather than being aberrant, might reflect the conservation of a physiological developmental potential, which is probably useful for tissue repair/regeneration [2,37,38,39].

Despite functional differences and the fact that they originate from distinct axial levels of the neural crest, the carotid body and the adrenal medulla are very much alike in tissue structure and cell types, and this similarity is maintained in the derived tumors. Furthermore, as demonstrated in several mammals, including humans, the adrenal medulla is also hypoxia-sensitive, particularly in the neonatal period of life [40]. All this suggests that the developmental and genetic pathways responsible for the growth and homeostasis of the carotid body and of the adrenal medulla could be very similar. In support of this hypothesis, studies based on genetic cell fate tracing and on genetic ablation of Schwann cell precursors in avian and mammalian models revealed that the adrenal medulla originates from neural crest-derived multipotent precursors with a glial phenotype (“Schwann cell precursors”), that migrate along the developing sympathetic nerve to the adrenal area to differentiate into postsynaptic neuroendocrine chromaffin cells [41,42]. Surprisingly, the conclusions of these publications, highly relevant to our understanding of PGL and pheochromocytoma, have yet to be incorporated into the mainstream pathological and molecular interpretation of PPGL tumorigenesis.

In fact, it is currently assumed that the phenotypic plasticity of PPGL cells is circumscribed within the neuroepithelial lineage, a theory backed by the neuroepithelial-specific loss of SDHB protein in the *SDH*-related PPGLs, which conforms to the widely accepted two-hit hypothesis of tumor suppressor genes [43,44,45,46]. This would imply that a uniquely neoplastic neuroepithelial cell population drives PPGL growth stimulating angiogenesis and gliogenesis from adjacent normal blood vessels and nerves. Thus, the vascular (endothelial and pericytic) and the glial (sustentacular) PPGL components are relegated to ancillary roles. Such a view is incongruent with the hypothesis that PPGL tumorigenesis could aberrantly recapitulate the histogenesis of the carotid body and of the adrenal medulla [47,48]. Thus, the origin(s) and the nature of PPGL remain undefined and controversial.

## 3. Molecular Heterogeneities Do Not Exclude a Developmental Model of Paragangliar Tumorigenesis

PPGLs have been linked to germline and/or somatic mutations in more than 20 genes considered tumor-initiators and/or -drivers [23,24,25]. PPGL tissues bear the distinguishable molecular signatures of these gene mutations, and, on such a basis, can be subdivided into at least three major molecular clusters [49]. The first and largest cluster, identified by pseudohypoxic signaling, is related to loss-of-function mutations that stabilize HIFA, either indirectly, via metabolic inhibition of the α-ketoglutarate-dependent dioxygenases, as in the case of mutations in the Krebs cycle genes encoding the SDH enzyme subunits (*SDHA*/*B*/*C*/*D*), the SDH assembly factor (*SDHAF2*), fumarate hydratase (*FH*) and malate dehydrogenase 2 (*MDH2*); or directly, via disruption of HIFA proteasomal targeting, as in the case of *VHL* and of the genes encoding the prolyl hydroxylases 1 and 2 (*EGLN1/2*). Additionally, gain-of-function mutations in *EPAS1*, encoding HIF2A, contribute to this cluster. Functionally, the pseudohypoxic cluster is characterized by steady HIFA signaling, even under normoxia, and by a cascade of downstream effects, including a metabolic shift towards glycolysis, impaired oxidative phosphorylation, production of reactive oxygen species, DNA and histone hypermethylation, inhibition of collagen maturation and activation of the EMT, which is the widely recognized driver of the migratory mesenchymal-like cell phenotype and of vasculoangiogenesis [50].

With the exception of the *VHL*-related PPGLs, frequently located in the adrenals, the pseudohypoxic cluster encompasses mainly noradrenergic extra-adrenal PGLs and is clinically important because it includes the *SDHB*/*FH*-related PGLs associated with higher metastatic potential and higher risk of disease multiplicity/recurrence [51]. The second cluster, designated the kinase signaling cluster, bears the molecular signature of aberrant PI3K/AKT and RAS/MAPK activation. Tumors in this cluster are mainly pheochromocytomas and have mutations in various genes involved in protein kinase signaling networks, including *NF1*, *KIF1B*, *MAX*, *RET, TMEM127*, *H-RAS*, *ATRX*, and, more rarely, *K-RAS* and *FGFR* [52]. PPGL-associated fusion genes involving *NGFR*, *BRAF*, or *NF1* also contribute to this group. Although lacking the central pseudohypoxic footprint, the kinase signaling cluster relies on a glycolytic and glutaminolytic switch, necessary for cell proliferation and survival, as well as for chromatin remodeling. Clinically, the PPGLs in this cluster do not display a particularly aggressive behavior, except those associated with *ATRX* mutations [52]. Finally, the third cluster, also mainly adrenal, designated the Wnt signaling cluster, is associated with mutations in the cold shock domain containing E1 (*CSDE1*) gene and with fusion genes involving the mastermind-like transcriptional coactivator 3 (*MAML3*). The PPGLs in this cluster tend to be hypomethylated and overexpress genes of the Wnt and Hedgehog pathways, known to play key roles in development [25]. Thus, the genomic landscape of PPGLs demonstrates clinically-relevant heterogeneity, but it is not granted that the distinctive molecular phenotypes entail substantial divergence in fundamental processes responsible for PPGL tissue development and growth. In fact, the molecular pathways defining the three major PPGL clusters are interrelated and participate in developmental processes [53,54,55]. Indeed, the relative uniformity of the organoid tissue organization of PPGLs suggests that different mutational backgrounds and molecular phenotypes converge on encouraging the aberrant activation of a single, pre-determined morphogenetic program that most likely retraces the developmental footsteps of paragangliar hyperplasia, as in the physiological model of the carotid body [37,56]. Furthermore, molecular phenotypes reflect microenvironmental interactions, which in complex tissues that contain cells of more than one type, like PPGLs, are likely modulated by the composition of the resident cell populations [57,58]. In this regard, PPGLs remain essentially faithful to their characteristic vasculo-neural architecture, but the extent to which the various vascular and neural cell types are represented in individual tumors, and their levels of differentiation, are variable [22,56]. Thus, the PPGL molecular clusters might reflect microenvironmental footprints, rather than differences in fundamental biological programs.

## 4. Ultrastructural and Immunomorphological Relationships Between the Vascular and Neural Compartments of Head and Neck Paragangliomas

In the past decade, we have tried to understand the relationships between the diverse PPGL cell types and to devise ways to capture the processes underlying PPGL development. Based on the characteristics of our patients, recruited at a skull base surgery center, we focused on HNPGLs, which mostly arise at the carotid bifurcation, in or around the jugular bulb, in the cervical tract of the vagus, or within the temporal bone. HNPGLs cause important morbidity and, when inoperable, are inevitably lethal [59].

We proceeded through sequential steps including: (1) analysis of the ultrastructural and immunomorphological relationships between the various resident HNPGL cell types; (2) identification of genes and molecular pathways common to HNPGLs; (3) localization of relevant protein products at the cellular and subcellular levels; (4) development and characterization of in vitro and in vivo models of HNPGL; and (5) use of such models, in conjunction with information derived from the preceding steps, to investigate HNPGL tissue development and evaluate the potential of specifically-targeted therapy [22,56]. None of the HNPGL cases recruited in our studies revealed evidence of metastasis, therefore our focus is on the reconstruction of the fundamental natural history of the disease, and not on factors linked to metastatic potential.

Using standard immunohistochemistry, classical electron microscopy (EM), and frozen section immunofluorescence (Figure 1), we confirmed that the endothelial, pericytic, glial, and neuroepithelial PGL cell types were clearly discriminated by specific markers (e.g., CD34, CD31, β2-microglobulin for endothelial cells; smooth muscle actin, S100, and GFAP for sustentacular cells; and chromogranin A and β3-tubulin for neuroepithelial cells). However, we also found that these allegedly distinct HNPGL cells coexpressed, to variable extents, markers associated with pluripotent mesenchymal stem-like state, vasculo/neurogenesis, and hypoxia (e.g., vimentin, nestin, CD44/HCAM, KIT/CD117, HIF2A, GLUT4, ZEB1, NOTCH1, DLK1, PDGFRA, VEGFR1/2) [22,56]. This was in agreement with flow cytometry, which highlighted within freshly-dissociated HNPGLs cell populations positive for stem-like mesenchymal cell markers (e.g., CD44/HCAM, CD73, CD90, CD105, and CD133). Further, the cells sorted for CD34 included subsets positive for stem (CD133, CD44/HCAM), neural (NCAM), or glial (GFAP) cell markers, suggesting pluripotency. A pluripotent potential was also consistent with the strong positivity of the endothelia for CD34, a sialomucin also expressed in mesenchymal progenitors and in gastrointestinal stromal tumors (GISTs), which co-occur with PGL in some *SDH*-related PGL syndromes [46,60], and for β2-microglobulin, a major histocompatibility complex (MHC) class I component associated with infection, the EMT, and cancer [61]. Furthermore, EM, that we extensively utilized, revealed aberrant features in the contiguous vascular (endothelial/pericytic) and neural (glial/neuroepithelial) HNPGL compartments [56], and highlighted widespread contacts between the pervasive dendritic processes of the sustentacular cells and the plasma membranes of the neuroepithelial cells, suggesting contact-mediated sustentacular nurturing [22]. Most notably, at the ultrastructural level, the HNPGL cell types demonstrated a gradient in mitochondrial alterations, limited to occasional swelling of the cristae in the endothelial, pericytic, and sustentacular cells, but striking in the neuroepithelial cells, where the mitochondria were massively increased in number, extremely swollen, and presented convoluted or disrupted cristae [56]. Additionally, the mitochondria tended to form tight perinuclear clustering, a subcellular redistribution connected to an oxidant-rich nuclear microenvironment that promotes hypoxia-induced transcription [62]. These aberrant mitochondria appeared to be incompatible with normal respiration. In fact, the mitochondrial membrane potential (ΔΨm) collapsed in the neuroepithelial PGL component relative to autologous normal adipose tissue, while the ΔΨm was only slightly decreased in the vascular component. This lineage-related pattern of mitochondrial alterations was found in all the HNPGLs analyzed, both mutated and unmutated in the *SDH* genes. However, larger mitochondria were significantly associated with the HNPGLs from *SDHB*/*C/D* gene mutation carriers [56].

## 5. Our Approach to the Study of Genes and Pathways Shared Among Head and Neck Paragangliomas

**Figure 2:**
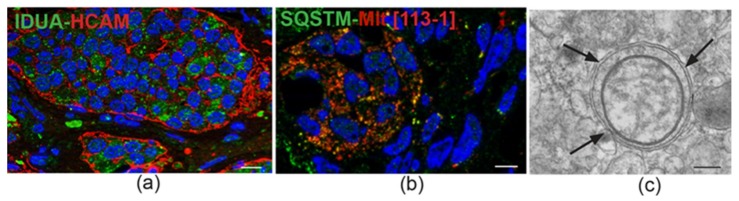
IDUA protein immunostaining and mitophagy in head and neck paraganglioma. (**a**) Immunofluorescence detects cytoplasmic IDUA protein labeling (green) in the neuroepithelial zellballen of paraganglioma. The zellballen are outlined in red by mainly peripheral labeling with antibody to HCAM/CD44, a surface stem cell marker that functions as a receptor for hyaluronan, a glycosaminoglycan degraded by the IDUA product (double immunofluorescence on semithin frozen section, bar = 10 µm). (**b**) Immunofluorescence highlights spots of colocalization (yellow) between anti-p62 antibody (Sequestosome-1, SQSTM, green) and anti-mitochondrial antibody 113-1 (ABCAM, red) (double immunofluorescence on semithin frozen section, bar = 10 µm). (**c**) Ultrastructural cross section of an autophagosome (indicated by arrows) containing a swollen mitochondrion, detected in the cytoplasm of a neuroepithelial paraganglioma cell (transmission electron micrograph, bar = 1 µm).

Back in 2013, we used high-density genome-wide copy number variation (CNV) analysis to identify HNPGL-related genes and pathways [22]. This analysis, then conducted on a pilot series of 24 tumors, including *SDH*-related and unrelated cases, versus matched blood, revealed in all cases a high level of chromosomal instability. A group of 104 genes, then mostly new to PPGL, was significantly over-represented among those affected by CNVs. We confirmed with orthogonal assays some of the most frequently amplified hits, including *IDUA* (4p16.3), *NOTCH1* (9q34.3), *JAG2* (14q32), *HES5* (1p36.32), *DVL1* (1p36), and *CTBP1* (4p16) [22]. Interestingly, *IDUA*, whose loss-of-function mutations are linked to type 1 mucopolysaccharidosis, a lysosomal storage disease (LSD) [63], showed the highest concordance for CN gains (*p* = 0.000002 by Fisher’s exact test). Notably, the HNPGL-derived *IDUA* gene sequences did not show mutations. By frozen section immunofluorescence, alpha-L-iduronidase, the *IDUA*-encoded enzyme, was strongly expressed in the neuroepithelial component of all tested PGLs, including cases not amplified at the *IDUA* locus (Figure 2) [22]. Alpha-L-iduronidase is necessary for the lysosomal hydrolysis of iduronic acid-containing glycosaminoglycans, such as dermatan sulfate and heparan sulfate, important microenvironmental cofactors of cell behavior in development and cancer, that act as receptors for viruses, exosomes, lipoproteins, and growth factors and control Fibroblast Growth Factor (FGF) and Sonic Hedgehog signaling [64,65,66]. While the above reported functions may be relevant to tumorigenesis, the link between *IDUA* and PGL can be better understood considering that mucopolysaccharidosis type 1 is associated with the accumulation of morpho-functionally altered mitochondria in neural cells, an alteration ascribed to impaired mitophagy due to alpha-L-iduronidase deficiency [67]. In fact, in carriers of loss-of-function *IDUA* mutations, mitochondrial clearance is compromised, leading to the intraneuronal accumulation of pathological mitochondria, characterized by low ΔΨm and swelling, loss of cristae, and vacuolation [67]. Contrariwise, in HNPGLs, alpha-L-iduronidase expression is high and the IDUA gene is unmutated [22], which suggests that the accumulation of dysfunctional mitochondria is due to primary factors and not to deficient clearance [56]. Indeed, high alpha-L-iduronidase expression might reflect upregulation of the mitophagic machinery, in response to the large and dysfunctional mitochondrial pool [68], a hypothesis supported by the frequent ultrastructural evidence of mitophagy in HNPGL neuroepithelial cells and by positivity of the mitochondria for LC3 and sequestosome (Figure 2).

## 6. Constitutive Notch Signaling in Head and Neck Paraganglioma

Bioinformatics analyses of tumor-derived gene databases are inherently biased toward better known pathways, which may divert attention from novelty. Nonetheless, it was notable that in our 2013 genome-wide CNV analysis of HNPGLs, “Notch signaling” stood out as the pathway with the highest statistical significance [22]. This pathway controls stem cell maintenance and binary cell fate specification in the vascular and parenchymal compartments, and directly affects nuclear and mitochondrial functions [69,70,71].

**Figure 3:**
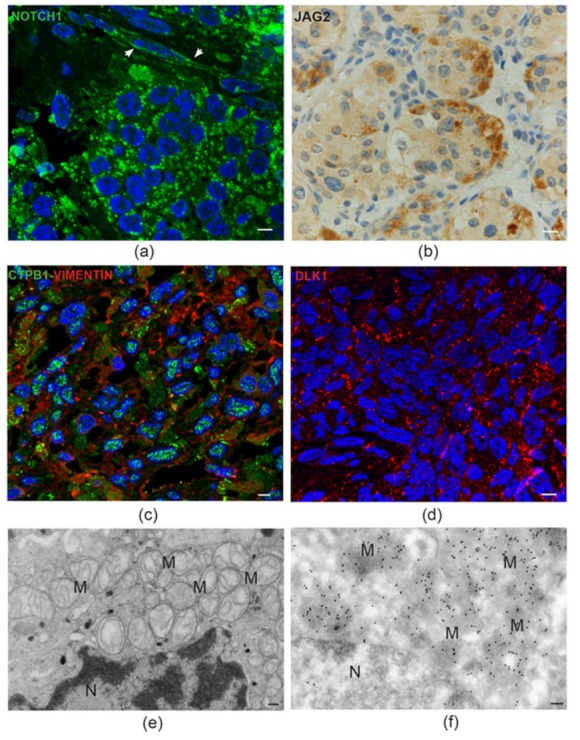
Notch pathway proteins in head and neck paraganglioma. (**a**) Semithin paraganglioma frozen section stained using immunofluorescence with an antibody that recognizes both membrane NOTCH1 and its active intracellular domain, NICD1 (green). In neuroepithelial cells, labeling is mainly concentrated in discrete cytoplasmic spots, suggesting mitochondrial localizations of NICD1. The adjacent endothelia (arrowheads) mainly reveal cell membrane NOTCH1 labeling (bar = 10 µm). (**b**) Immunohistochemical staining for the NOTCH ligand JAG2 is intense at the periphery of the neuroepithelial cell clusters, a typical location of the sustentacular cells (standard avidin-biotin immunoperoxidase counterstained with hematoxylin and eosin, bar = 10 µm). (**c**) Double immunofluorescence on semithin paraganglioma frozen section highlights punctate CTBP1 nuclear labeling in most cells. Red labeling identifies cells staining positive for vimentin, a mesenchymal marker (double immunofluorescence on semithin frozen section, bar = 10 µm). (**d**) Punctate membrane staining pattern (red) of the atypical NOTCH ligand DLK1 in paraganglioma cells (immunofluorescence on semithin frozen section, bar = 10 µm). (**e**) Electron micrograph of a neuroepithelial (“chief”) paraganglioma cell showing the accumulation of swollen mitochondria with disrupted cristae (M) next to the envelope of the nucleus (N) (bar = 1 µm). (**f**) Immunoelectron microscopic view of a similar ultrastructural field, showing dense NICD1 labeling of the perinuclear mitochondria with gold particles (ultrathin frozen section immunoelectronmicroscopy, bar = 1 µm).

The statistical emergence of Notch signaling rested on five Notch signaling-related genes targeted by recurrent amplifications [22]. These included *NOTCH1*, prototype of the NOTCH receptor family, *JAG2*, a NOTCH ligand linked to vasculogenesis and the EMT, *HES5*, a NOTCH1-activated transcriptional repressor involved in neural stem cells induction [72,73], *DVL1*, hub of the interactions between Notch and Wnt signaling [74], and *CTBP1*, a transcription regulator sensitive to the reduced form of nicotinamide-adenine dinucleotide (NADH), that in melanoma cells links NOTCH signaling to the drop of the intracellular NAD+:NADH ratio caused by aerobic glycolysis [75,76]. NOTCH1 signaling is mediated by the NOTCH1 intracellular domain (NICD1), released by proteolysis of transmembrane NOTCH1 after ligand-induced activation, which relocates to the mitochondria and to the nucleus. In the mitochondria NICD1 inhibits BAX and deregulates complex I, an effect that could contribute to explain the deregulation of complex I activity reported in the *SDH*-mutated PGLs [77,78]. In the nucleus, NICD1 forms a transcriptional regulatory complex with Suppressor of Hairless and Mastermind, which prevents the expression of cell differentiation factors and mediates the HIFA-induced metabolic changes resulting in the Warburg effect [79,80]. Importantly, NOTCH and HIFA signaling are linked in a positive loop: hypoxia promotes NOTCH activation, and NOTCH signaling upregulates HIF2A, the driver of the pseudo-hypoxic phenotype [81]. As investigated using immunohistochemistry, immunofluorescence, and cryo-immuno-EM (Figure 3), the protein products of the top-amplified NOTCH1-related genes were highly expressed in all the PGLs analyzed, independently of CNV status at the respective loci and of presence or absence of germline *SDHx* mutations [22]. However, for some of these proteins, the levels and the subcellular localizations of the immunostaining varied with cell type. JAG2 was mainly expressed in the sustentacular cells, including their dendritic processes, which establish multiple contacts with the neuroepithelial cells [22]. Membrane NOTCH1 was strongest in the endothelial and sustentacular cells, while mitochondrial and nuclear NOTCH1 was more conspicuous in the neuroepithelial component, where the mitochondria are severely altered and contiguous to the nuclear envelope (Figure 3), suggesting a mitochondrial role in the nuclear delivery of NICD1 [22,56].

**Figure 4:**
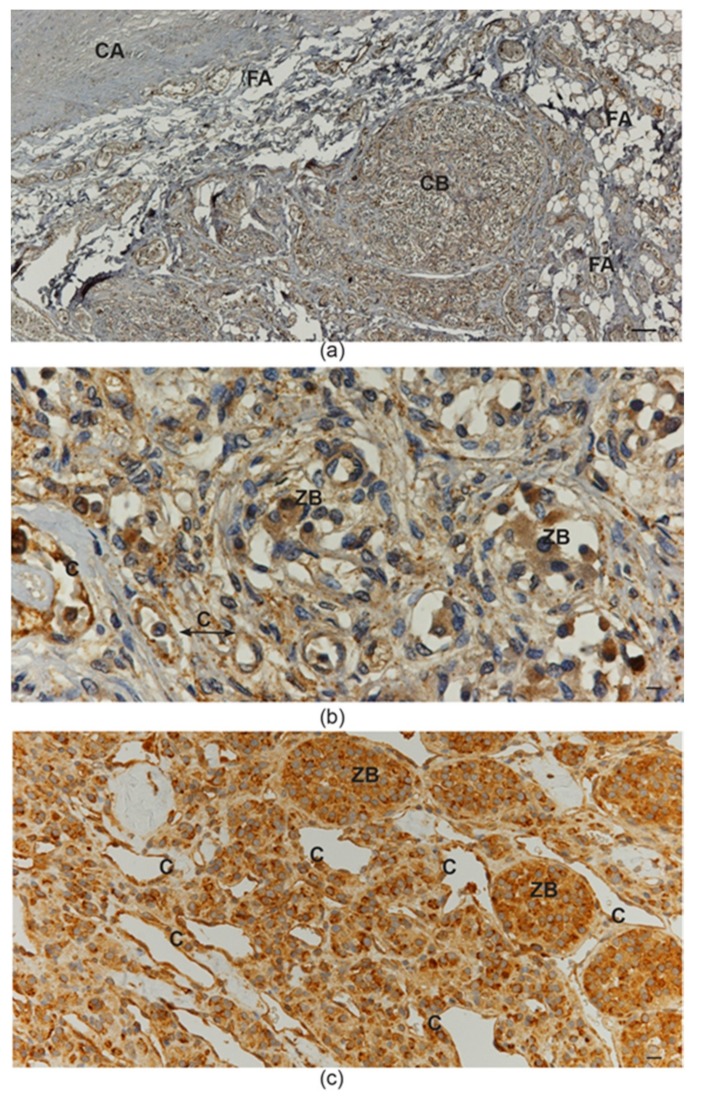
NOTCH1 protein immunostaining in normal carotid body and in carotid body paraganglioma. (**a**) Low-power view of the fibroadipose tissue located at the carotid bifurcation, which contains paragangliar tissue immunostained (brown) with NOTCH1 antibody (avidin-biotin immunoperoxidase counterstained with hematoxylin and eosin, CA: carotid artery; FA: fibroadipose tissue; CB: carotid body; bar = 100 µm). (**b**) High-power view of the carotid body tissue immunostained (brown) with NOTCH1 antibody. Both capillary endothelia and neuroepithelial cells within “zellballen” are immunostained (avidin-biotin immunoperoxidase counterstained with hematoxylin and eosin, C: capillaries; ZB: “zellballen”; bar = 20 µm). (**c**) Paraganglioma tissue immunostained (brown) with NOTCH1 antibody. Ectatic capillaries and “zellballen” are immunostained (avidin-biotin immunoperoxidase counterstained with hematoxylin and eosin, C: capillaries; ZB: “zellballen”; bar = 25 µm).

The strong association of the NOTCH signaling pathway with HNPGLs may not simply reflect a pathological condition. In fact, we recently performed a limited immunohistochemical study on scarce paraffin-embedded sections of a warm autopsy-derived normal human carotid body, which revealed NOTCH1 immunostaining of the vascular and neural tissue components, less intense but similar to that observed in the HNPGLs (Figure 4), hinting to a physiological role of NOTCH1 signaling in paraganglia. In this respect, it is intriguing that complex I deregulation is necessary for normal carotid body function [82]. All this may exemplify the repurposing of developmental and morphogenetic pathways in HNPGL tumorigenesis.

## 7. Patient-Derived Head and Neck Paraganglioma Cultures Exhibit a Multipotent Mesenchymal-Like Phenotype

The lack of human PPGL-derived cell lines may reflect the difficulty of maintaining neuroepithelial PPGL cells under culture conditions. In fact, these cells are thought to be the unique neoplastic component of the tumor tissue [44,45,46]. However, a developmental origin of PPGL would rather be consistent with a multipotent stem/progenitor phenotype of PPGL cells in culture. We generated several primary and at least four lentivirus-immortalized HNPGL cell cultures [56]. Both the primary and the immortalized cultures demonstrated quite homogeneous flow cytometric profiles, positive for classic mesenchymal markers (CD73, CD90, CD105), for embryonic and neural stem cell markers (SOX2 and nestin), and for GFAP and PDGFRA. Such characteristics were common to cultures from PGLs with and without constitutional *SDH* gene mutations. Immunofluorescence confirmed expression of the immature mesenchymal, hypoxic, and vascular/neural markers shared by the diverse tissue components of the PGLs of origin (e.g., vimentin, nestin, CD44/HCAM, KIT/CD117, HIF2A, GLUT4, ZEB1, NOTCH1, DLK1, PDGFRA, and VEGFR1/2). In tridimensional foci, randomly formed under standard culture conditions, and in neurospheres, formed under non-adhesive conditions, the outer shell of cells exposed to the medium was vimentin-positive and nestin-negative, while the reverse occurred in the putatively hypoxic inner cell core. In matrigel, which allows tridimensional growth, the cells readily generated pseudovascular networks expressing CD34 together with DLK1 and PDGFRA, known components of the molecular mechanisms involved in vasculogenesis [56]. Notably, both the primary and the immortalized HNPGL cells had normal tubular mitochondria with high ΔΨm, implying normal respiratory functions. However, mitochondrial alterations similar to those found in the HNPGLs of origin where observed in cell-derived xenografts (CDXs) formed after subcutaneous transplantation into nude mice [56]. This indicates that the dysfunctional mitochondria observed in the neuroepithelial HNPGL component are not exclusively determined by genetic alterations, but develop under the influence of microenviromental and differentiation-related factors.

## 8. The microRNA-200s and -34s Modulate NOTCH1, ZEB1, and PDGFRA Levels in Paraganglioma

We addressed the hypothesis that microRNAs (miRNAs) could contribute to the establishment of an immature mesenchymal phenotype in HNPGL. We therefore compared the miRNA profiles of HNPGLs (13 independent tumors) to those of pools of normal Jacobson’s nerves (JN), a parasympathetic nerve that is a frequent site of origin of tympanic HNPGL [22]. JN has the unique advantage of being recoverable, as it must be removed during petrosectomy, and has never been used as a reference tissue for HNPGL by other authors. Genome-wide miRNA expression profiling on the Illumina platform, validated using reverse transcription quantitative PCR (RT-qPCR), revealed that 16 miRNAs were significantly downregulated in the HNPGLs, and only three were significantly upregulated [22]. Notably, the miRNAs most significantly downregulated included the miR-200a,b,c, which inhibit the EMT and promote cell differentiation and senescence by targeting the E-cadherin transcriptional repressors *ZEB1* and *ZEB2* [83,84], and the miR-34b, a mediator of TP53 function [85]. Enforcing Mir expression via transfection in the SH-SY5Y neuroblastoma cell model, or via lentiviral infection in our primary or immortalized HNPGL cells, we proved that the miR-200b,c and the miR-34b directly target NOTCH1 and that miR-200a indirectly influences the NOTCH pathway [22]. We also confirmed in the same models that the miR-200a,b/429 cluster strongly reduces both PDGFRA and ZEB1 RNA and protein levels, while the miR-34b,c cluster strongly downregulates PDGFRA, but not ZEB1 [56]. Reintroduction of these miRs in PGL cells was followed by cell death accompanied by upregulation of BAX protein expression, indicating activation of an apoptotic response [22,56]. In conclusion, the loss of miR-200 and miR-34 family members influences the molecular and cellular HNPGL microenvironment by promoting the upregulation of key EMT- and mesenchymal-related genes. This may be of translational relevance: in fact, PDGFRA, together with KIT/CD117, also expressed in HNPGLs [56], are key targets of imatinib, a drug highly effective in the prevention and treatment of GISTs [86,87]. Furthermore, the NOTCH pathway and ZEB1 are major inducers of chemo- and radio-resistance [88].

## 9. The Lesson of the Xenograft Models

**Figure 5:**
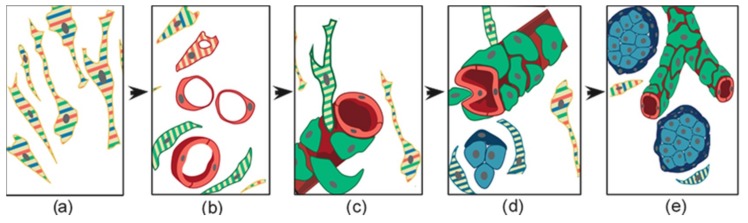
Schematic representation of the histogenesis of head and neck paraganglioma, based on patient- and cell-derived xenograft models. The thin elongated stem-like cells (**a**), stabilized and expanded in paraganglioma cell cultures, co-express (stripes) multipotent stem/progenitor cell markers. In vivo, (**b**) these cells grow on autonomously synthesized extracellular matrix, develop intracytoplasmic vacuoles of increasing size (cells with red stripes only), and coalesce (uniformly red cells), giving rise to endothelial tubes (vasculogenesis via cytoplasmic hollowing). The endothelium then recruits adjacent stem-like cells (cells with green stripes only), which, after contact with the abluminal endothelial membranes (**c**), differentiate into mural cells (uniformly green). (**d**) The panel outlines two remarkable consequences of mural stabilization. First, mural impingement results in intraluminal endothelial intussusceptions, which divide the flow, giving rise to Y-shaped vascular ramifications (intussusceptive angiogenesis, a process detectable only with whole-mount confocal microscopy and/or transmission electron microscopy, as used in our study [56]). Secondly, vascular stabilization results in perivascular deposition of collagen IV [56], which supports the development of cell clusters with neural phenotype (cells with blue stripes only, then uniformly blue). As shown in (**e**), these clusters develop into “zellballen”-like neuroepithelial nests (uniform light blue), bound by spindle-shaped glia-like cells (uniform dark blue). Notably, while mesenchymal paraganglioma stem-like cells have normal mitochondria, paraganglioma tissue organization is associated with increasing mitochondrial dysfunction (swelling and loss of membrane potential), culminating in the neuroepithelial component. Original art by Giulio Pandolfelli, adapted and modified from Verginelli et al., 2018 [56].

The formation of patient-derived tumor xenografts (PDXs) in immunosuppressed mice is predicted to depend on cells that survive ischemic necrosis during the prolonged avascular phase that follows surgery and lasts until a new vascular network links the PDX to the murine circulation [56]. To investigate the cells that survive in PGL tissue after devascularization, we analyzed ex vivo-cultured PGL samples corresponding in size to those xenografted in mice. Light microscopy and EM showed extensive coagulative necrosis by day 10 post-surgery, but also revealed areas recolonized and remodeled by endogenous smooth muscle actin/vimentin-positive, collagen-producing mesenchymal-like cells showing phagocytic capability, plausibly useful for the recovery of nutrients from necrotic tissue. These cells were similar to those found at an early phase of subcutaneous PDX formation in immunodeficient mice (3 weeks) [56]. We further analyzed the ultrastructural and light microscopic morphology of 90 PDX samples from different HNPGL patients, all implanted subcutaneously into the flanks or neck. The overall take-rate at 4.5–10 months was high (89%) and unrelated to *SDH* mutation carrier status. The PDX tissues, including the vasculature, proved to be of human origin, as demonstrated by mitochondrial DNA analysis and immunoreactivity with antibodies recognizing human, but not mouse, antigens [56]. Permeation with intracardiacally-injected India ink solution demonstrated connections to the murine circulation. Typically, given the transplantation sites, the PDXs infiltrated the cutaneous branches of the dorsal spinal nerves, imitating the perineural growth typical of HNPGL. However, despite the locally aggressive behavior, the PDXs never exceeded ≈6 mm in maximum diameter, a size consistent with the slow growth of HNPGLs in patients [56]. EM and thin section immunofluorescence revealed that PDX tissue organization initiated with a vasculogenic process, schematized in Figure 5, which led to the formation of endothelial tubes [56]. Such tubes originated from the self-assembly of individual endothelial precursors, that first developed intracytoplasmic lumina through cytoplasmic vacuolization (cell hollowing), as in HUVECs and in drosophila and zebrafish embryos [89,90]. The lumenized tubes were positive for human β2-microglobulin, CD31, and CD34, as HNPGL endothelium, and defined a perivascular niche that attracted mesenchymal-like, smooth muscle actin-positive cells [56,91]. The adherence of these cells to the abluminal endothelial cell membranes was associated with dichotomic branching of the endothelial tube, a morphology pointing to intussusceptive angiogenesis, a form of nonsprouting angiogenesis that allows the rapid bifurcation of neoformed vasculature via endothelial invaginations [56,92]. Notably, in this process, membrane NOTCH1 was uniquely present on the abluminal endothelial membrane, in contact with adhering perivascular cells strongly positive for DLK1, a HIF-induced non-canonical NOTCH antagonist known as a cancer pericyte antigen [56,93]. These DLK1-positive cells also coexpressed smooth muscle actin and PDGFRA, assuming the ultrastructural and immunophenotypic characteristics of mural cells or pericytes [56]. The initial vasculo-angiogenic network was supported by autonomously synthesized collagen I, an EMT-related collagen [56,94]. With the formation of structured vessels, supported by mural cells or pericytes, the perivascular matrix was enriched with collagen IV, a key component of basement membranes [56,95]. Such microenvironmental modification was associated with the development of neuroepithelial-like cell clusters, often encircled by glia-like spindle cells (Figure 5).

**Figure 6:**
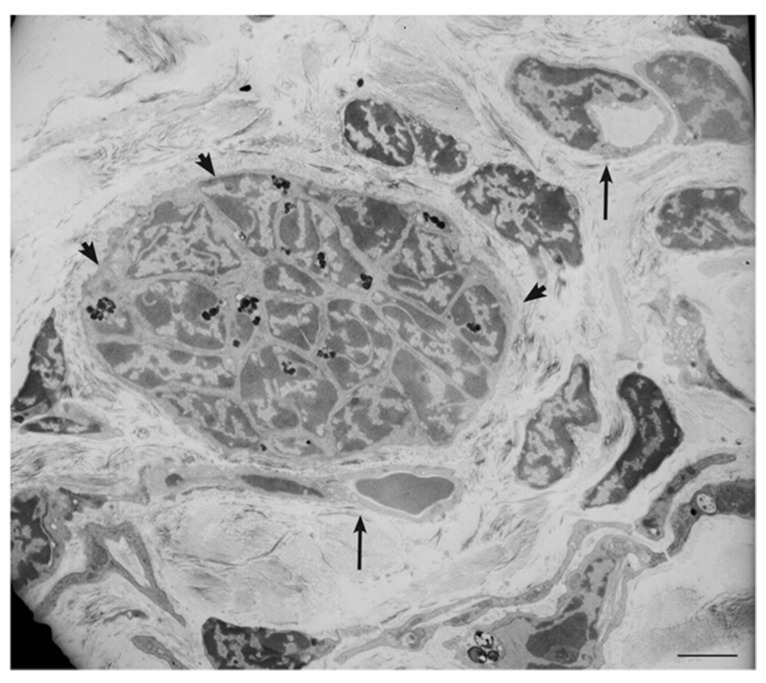
Ultrastructural view of paraganglioma xenograft tissue. The electron micrograph, derived from a xenograft obtained by subcutaneous injection of an immortalized tympano-jugular paraganglioma cell line (PTJ64i), shows a tight neuroepithelial-like cell cluster (arrowheads, dark spots are lipofuscins) in the context of a vasculogenic tissue revealing endothelial-like cells with cytoplasmic hollowing and capillary-like structures (arrows) (bar = 5 µm).

These cell nests revealed positivity for the immature mesenchymal, hypoxic, and vasculoneurogenic markers found in the neuropithelial component of PGLs (e.g., vimentin, nestin, CD44/HCAM, KIT/CD117, HIF2A, GLUT4, ZEB1, NOTCH1, DLK1, PDGFRA, and VEGFR1/2), but lacked advanced neuroendocrine markers, such as chromogranin A and synaptophysin. Ultrastructurally, most PDX cells, and particularly those of the neuroepithelial-like nests, exhibited hyperplastic and swollen mitochondria with vesicular or disrupted cristae, as in the neuroepithelial cells of the HNPGLs of origin [56]. Cell-derived xenografts (CDXs) were similarly obtained after subcutaneous injection of an immortalized HNPGL cell line (PTJ64i) into immunodeficient mice. At 45 days from transplantation the cells formed flat red-brown patches of 4–6 mm in diameter that, as the PDXs, comprised a vasculo-angiogenic network supporting nests of neuroepithelial-like cells (Figure 6) [56]. As noted before, the CDX cells developed hyperplastic and swollen mitochondria, resembling those of the neuroepithelial HNPGL component.

Overall, despite their slow development and small size, our HNPGL xenograft models support the view that HNPGL histogenesis does not depend on neurogenesis and ancillary sprouting angiogenesis, but on primary vasculogenesis of the embryonic type, followed by neurogenesis [56]. This is in agreement with the physiological model of the hyperplastic carotid body [36] and emphasizes the link between HNPGL development and embryogenesis, where vasculogenesis precedes and guides histo/organogenesis, a sequence recapitulated in postnatal tissue regeneration [96,97].

## 10. Imatinib Blocks HNPGL Cell Growth and Inhibits Xenograft Formation

The evidence that HNPGLs express PDGFRA and KIT/CD117, the receptor tyrosine kinases targeted by imatinib, brought us to test the effects of this drug on our HNPGL models [56]. At low dose (10 μM), imatinib inhibited the growth of four HNPGL cell cultures tested, three primary and one immortalized, that are representative of *SDH*-related and unrelated HNPGLs. Imatinib treatment was followed by global protein dephosphorylation, downregulation of the ZEB1, PDGFRA, and PDGFRB proteins, upregulation of Beclin 1, core component of the autophagy machinery, activation of the caspases 3/7 and induction of BAX. Treatment was also followed by mainly upward variation in the levels of miR-200a/b/c and miR-34b/c, consistent with the observed downregulation of the ZEB1 and PDGFRA proteins. Imatinib also significantly prevented CDX formation in immunodeficient mice. In this case imatinib (50 mg/kg for 20 days, then 16.6 mg/kg for 20 additional days) was given by intra-peritoneal injection, starting at 72 hours from the subcutaneous inoculation of the immortalized HNPGL cell line PTJ64i. Only 2 CDXs were detected at 10 heterotransplant sites in the imatinib-treated group versus 11 at 12 sites in the control group (*p* = 0.0015). Furthermore, the 2 CDXs found in the treated mice contained only disorganized or apoptotic cells with diffuse evidence of autophagic vacuoles [56], suggesting that imbalanced autophagic flux contributed to imatinib-induced growth arrest and apoptosis, as previously demonstrated by other authors in several mammalian cell types [98].

## 11. Conclusions

This perspective review challenges the prevalent view postulating that PPGLs are exclusively neuroendocrine tumors [44,45,46]. In fact, we propose that HNPGL arises from mesenchymal stem-like cells with vasculo-neural differentiation potential, in keeping with the neural crest derivation of paraganglia [8,41,42] and with the hyperplastic carotid body model, where the vascular and the neural components arise from resident stem-like cells retaining mesectodermal differentiation potential, reactivated by chronic hypoxia [36]. In support of this, retention of mesenchymal markers is evident in all the distinct HNPGL tissue components. Furthermore, mesenchymal stem-like cells persist in HNPGL tissue, and after damage, might be regenerated via EMT from more differentiated vascular and/or neural cells [56].

HNPGL xenografts can be viewed as attempts to HNPGL regeneration after devascularization. In patients, tumor regeneration after embolization, radiotherapy or chemotherapy could follow in the same footsteps. In essence, our xenograft models, based on tumors related and unrelated to *SDH* gene mutations, show that the vascular and neural HNPGL tissue components sequentially emerge following an endogenous developmental program [56]. Primordial endothelial tubulogenesis seems to be the earliest histogenetic event, as in embryonic development. This process is complemented by angiogenesis, which does not follow the well-known sprouting model, but exploits endothelial intussusception [56], a stochastic intravascular process of dichotomic branching, mediated by largely unexplored paracrine and contact-mediated signals [99,100]. Interestingly, during the development of the early vasculo-angiogenic network, the interaction between endothelial and pericytic/mural cells involves compartmentalized expression of the NOTCH1, PDGFRA, and DLK1 proteins [56,101]. NOTCH1 is localized on the abluminal endothelial membrane, i.e., the original plasma membrane of the mesenchymal-like cell that, by vacuolization, differentiates towards the endothelial lineage (Figure 5), whereas PDGFRA and the atypical NOTCH ligand DLK1 are expressed by the smooth muscle actin-positive periendothelial cells engaged in pericytic/mural differentiation, which physically interact with the NOTCH1-labeled endothelial membranes [56].

The dependence of HNPGL histogenesis on a primordial vasculo-angiogenic phase provides a rationale for targeted preventive and therapeutic interventions, which, however, would require a better understanding of the molecular mechanisms underlying endothelial tubulogenesis and intussusceptive angiogenesis. Nonetheless, imatinib, which targets the recruitment of PDGFRA-positive mural/pericytic cells necessary for the stabilization of endothelial tubes [87,91], strongly prevented CDX formation in our murine model. Given that tumor maintenance and tumor development are distinct phenomena [102], this may not be translated into the conclusion that imatinib could effectively target structured HNPGL tissue in patients, but raises the intriguing possibility of whether this drug could be given in a preventive setting after surgery, embolization, or radiotherapy in order to reduce the risk of HNPGL regeneration. This important question remains to be addressed with appropriate study designs. Interestingly, vasculo-angiogenesis, as well as the EMT, are predicted to be negatively controlled by the miR-200a,b,c and by the miR-34b [103,104], which were significantly downregulated in our HNPGLs relative to our normal parasympathetic neural control, JN [22]. *ZEB1*, *NOTCH1*, and *PDGFRA* are coordinately targeted by these miRs, thus the high levels of the relative protein products in our HNPGLs can be at least partly explained by the loss of miRNA-mediated regulation [22,56]. Additionally, the significant amplification of NOTCH pathway genes, demonstrated by us for *NOTCH1*, *JAG2*, *HES5*, *DVL1,* and *CTBP1*, must concur with the constitutive upregulation of NOTCH1 signaling in HNPGLs. This might contribute to link cell fate decisions to metabolism via the coordinated transcriptional effects exerted by NICD1, at the mitochondrial and nuclear levels, and by nuclear CTBP1, a sensor of the NAD+/NADH ratio. NOTCH1 signaling is likely fundamental not only for the development, but also for the homeostasis of HNPGL. In fact, neuroepithelial dependence on NOTCH1 signaling via JAG2, delivered by sustentacular cells, may account for the extensive interactions between dendritic sustentacular processes and neuroepithelial cells, where BAX inhibition and complex I deregulation, contributed by NICD1, might help to sustain dysfunctional mitochondria. Furthermore, in connection with the upregulation of ZEB1 and the EMT, NOTCH signaling is predicted to promote resistance to chemo/radiotherapy and to antiangiogenic agents, which are major problems in PPGL therapy [105,106,107,108,109].

Widespread mitochondrial alterations, that correlate with neuroepithelial differentiation and loss of ΔΨm, implying glycolytic dependence, are a key ultrastructural feature basically common to HNPGLs, again independently of their genetic backgrounds [56]. Such alterations, not present in our cultured mesenchymal-like HNPGL cells, are acquired after in vivo transplantation, which links these mitochondrial changes to microenvironment-related factors. In this regard, the role of complex I deregulation is probably central, but is still debated. In fact, several studies reported loss of complex I activity in PPGLs and in *SDHB*-mutated cell models [77,110,111], whereas Pang and coworkers recently found an upregulation of complex I, accompanied by a strengthened NAD+ metabolism, in *SDHB*-mutated PPGLs [78]. The latter finding suggests that complex I could compensate for the primary loss of complex II activity characteristic of *SDH*-mutated PPGLs, an effect of potential relevance in the clinical setting, as it could account for differential sensitivities to chemotherapeutic agents [78]. The question is clearly open, and in our opinion, could be addressed taking into account the microenvironmental contexts. In fact, viewing each neuroepithelial PPGL cell and each PPGL tissue as an ecosystem, it could be hypothesized that complex I activity is balanced in the mitochondrial populations to meet specific metabolic needs that contribute to the homeostasis of stressed neuroepithelial cells in the variable tumor microenvironment. This is an area that is currently addressed in our laboratories in Chieti and Stockholm.

To sum up, we challenge the view of PGL as the prototype of “Warburg tumors” [43]. In this perspective, PGL cells would be constrained into a pre-defined role conforming to “classic” two-hit or multiple-hit, gene-centered paradigms, where random genetic and epigenetic changes, driven by “selective pressures,” result in the emergence of heterogeneous and uncoordinated clonal tumor subpopulations. Instead, we believe that, regardless of genetic heterogeneities, HNPGL tumorigenesis essentially adheres to a finalized and pre-defined histogenetic program, most likely retracing the footsteps of carotid body histogenesis [10,35,36]. Our findings likely bear on the development of PPGLs in general and could open up to a new understanding of the disease.
